# The Landscape of Microbial Composition and Associated Factors in Pancreatic Ductal Adenocarcinoma Using RNA-Seq Data

**DOI:** 10.3389/fonc.2021.651350

**Published:** 2021-05-31

**Authors:** Dong Yu, Tengjiao Wang, Dong Liang, Yue Mei, Wenbin Zou, Shiwei Guo

**Affiliations:** ^1^ Center of Translational Medicine, Second Military Medical University, Shanghai, China; ^2^ Shanghai Key Laboratory of Cell Engineering, Shanghai, China; ^3^ Department of Gastroenterology, Changhai Hospital, Second Military Medical University, Shanghai, China; ^4^ Department of General Surgery, Changhai Hospital, Second Military Medical University, Shanghai, China

**Keywords:** PDAC (pancreatic ductal adenocarcinoma), tumor tissue, microbial composition, biological factors, PERMANOVA (permutational multivariate analysis of variance) test, Wilcox test

## Abstract

Recent research studies on interrogation of the tumor microbiome (including bacteria, viruses, and fungi) have yielded important insights into the role of microbes in carcinogenesis, therapeutic responses, and resistance. Once thought to be a sterile organ, a number of studies have showed the presence of microbes within this organ in PDAC status. A microbiome–pancreas axis for PDAC (pancreatic ductal adenocarcinoma) carcinogenesis is proposed. However, the microbial composition of localized PDAC tissue is still unclear. The associations between microbiome and PDAC reported in previous studies were detected in an indirect way, which mostly used samples from stool, oral saliva, and intestinal samples. This study integrated 582 samples derived from PDAC tissues across four datasets and presented a landscape of tumor microbiome at the genus level in PDAC based on remining of RNA-Seq data. On average, there are hundreds of genera distributed in the PDAC tissue, and dozens of core microbiota were identified by PDAC tissue. The pan-microbiome of PDAC tissue was also estimated, which might surpass 2,500 genera. In addition, sampling sites (stroma *vs*. epithelium) and tissue source (human tissue *vs*. PDX) were found to have great effects on the microbial composition of PDAC tissue, but not the traditional risk factors (sex and age). It is the first study to systematically focus on exploring the microbial composition of PDAC tissue and is helpful to have a deep understanding of tumor microbiome. The identified specific taxa might be potential biomarkers for follow-up research studies.

## Introduction

Pancreatic ductal adenocarcinoma (PDAC) is one of the most aggressive cancer in the world with an annual incidence increased by 2.3 times from 1990 to 2017 (1). The incidence and mortality of PDAC are nearly equal, with a very poor 5-year survival rate of 9% (2). In the past, various studies were conducted to dissect the omics landscape to explore mechanisms of carcinogenesis, diagnosis, and therapy. The kinds of subtypes were revealed by integrated multi-platform analysis, which have prognostic significance and therapeutic implications (3, 4). However, there is still no effective molecular markers for early screening, diagnosis, and treatment. A novel approach to PDAC is desperately needed.

Recently, more studies focus on the role of tumor microbiome (including bacteria, virus, and fungi) in carcinogenesis, therapeutic responses, and resistance. Once thought to be a sterile organ, a number of studies have showed the presence of microbes within this organ in PDAC status (5, 6). Compared with normal pancreatic tissue, a 1,000-fold increase of bacteria in intrapancreatic was identified in PDAC patients (6). *H. pylori* was found to colonize the pancreas and may associate with the malignant potential of PDAC (7, 8). The localized *Fusobacterium* in PDAC tissue was identified as an independent prognostic factor for significantly shorter survival (9). A recent study found that the tumor microbiome and alpha diversity between long term survival patients and short term survival patients have significant differences, and an intra-tumoral microbiome signature was further identified to be predictive of long term survivorship in both discovery and validation cohorts (10). Rogier et al. found the co-occurrence and enrichment of oral bacterial taxa including *Fusobacterium nucleatum* and *Granulicatella adiacens* in cyst fluid from iPMn with high-grade dysplasia, which suggests an enormous role of oral bacterial taxa in cystic procedures to pancreatic cancer (11). These studies indicated the important roles of intrapancreatic microbiota in tumor progression. In addition, the intrapancreatic microbiota in pancreatic cancer could confer resistance to gemcitabine by breaking down gemcitabine into an inactive form *via* a specific isoform of the enzyme cytidine deaminase (12). In a word, microbiota do exist in the microenvironment of pancreatic tumor tissue and play important roles in tumor carcinogenesis and progression.

However, there is still a lack of systematic understanding of microbial composition in PDAC as we do in gut microbiome. For example, how many microbial taxa are there in the PDAC tissue samples? Is it in a stable state or dynamic state as the disease progresses? Or is there a gender difference? Are there differences in microbial composition within different anatomical regions of PDAC tissues? To answer these questions, we need to delve deeper into the microbiome of PDAC. Most pointedly, most of the above-mentioned studies detected the associations between microbiome and tumor in an indirect way, which used the samples from stool, oral saliva or intestinal tissues. We need to directly explore the true microbial composition based on localized PDAC tissue samples.

Over the past decades, the omics data, mainly whole genome sequencing and whole transcriptome sequencing data, has been greatly increased with the popularization of Next-generation technology. In the past few years, a number of studies found that microbial reads existed in the conventional cancer-omics data (13–18). A series of bioinformatic pipelines, including Pathseq (19), CaPSID (20), PathoScope (21), Kraken2 (22), and Shogun (23) were developed to mine the microbial information hidden in the sequencing reads. In these studies, most of the results were then validated by 16S rRNA sequencing or qPCR method, which indicate the methods’ feasibility and reliability. The most recent study provided a landscape of cancer microbiomes in 32 cancer types based on TCGA datasets, and ML models with high performance were then trained to discriminate between and within types and stages of cancers (16). In this perspective, it provides a new idea and method for exploring the microbial composition of localized tumor tissues.

To our knowledge, no studies have yet characterized the microbial composition of PDAC tissues using RNA-Seq data across multiple datasets. Here we have investigated four RNA-Seq datasets with 582 PDAC tissue samples from four irrelevant studies. The microbial profiles of each dataset were separately generated and then integrated for analysis. Several biological factors were found to have great or no effects on the microbial composition of tumor tissues. The results would provide important implications for the follow-up tumor microbiome-related research studies.

## Methods

### Dataset Selection

We conducted a database retrieval with key words “pancreatic ductal adenocarcinoma” and “expression profiling by high throughput sequencing” in NCBI GEO database. Three datasets (ERP022034, SRP096338, SRP197641) with all tumor samples were finally screen out for further analysis. The raw fastq files and meta files were downloaded. The microbial composition of PDAC samples in TCGA dataset was also downloaded from the ftp site (ftp://ftp.microbio.me/pub/cancer_microbiome_analysis/). The missing information of meta files were filled in according to the corresponding literatures as listed in **Table 1**. Two additional datasets (GSE105083, GSE74927) were also downloaded as positive and negative references to validate the reliability and flexibility of the following computational framework.

**Table 1:** An overview of meta information of four PDAC datasets.

Study	ERP022034	SRP096338	SRP197641	TCGA-PAAD
DataSubmittedLab	AROS Applied Biotechnology A/S (Aarhus, Denmark).	Klinikum rechts der Isar	The University of North Carolina at Chapel Hill	University of North Carolina
**Sequencing Platform**				
HiSeq	30	214	66	147
NextSeq	/	/	125	/
**Library Layout**				
single	/	214	66	/
paired	30	/	125	147
**Experiment Strategy**				
NuGENExome	/	199	/	/
TruSeqExome	/	15	55	/
TruSeqmRNA	30	/	136	/
**Sample Type**				
FF	/	/	70	/
FC	30	214	/	/
FFPE	/	/	7	147
Biopsies	/	/	114	/
**Tissue Source**				
Human Tissue	/	214	163	147
PDX	30	/	28	/
**Sampling Site**				
stroma	/	128	/	/
epithelium	/	71	/	/
bulk	30	15	191	147
**Sex**				
male	16	/	/	79
female	14	/	/	68
**Age**				
<=65	/	/	/	75
>65	/	/	/	72

“/” means NA, which indicates that the dataset does not possess the characteristic.

### Computational Framework for Microbial Detection

Raw fastq files were first processed for quality estimation using software FASTQC and then trimmed for quality control using software TRIMOMMATIC. The sequencing reads after quality control were mapped to the human reference databases to remove human reads. The human reference database consists of five parts: the latest human genome reference GRCh38, Immuno Polymorphism database (IPD) containing highly variable sequences of the human major histocompatibility complex (MHC), NCBI UniVec containing cloning vector sequences, Gencode (human v32) containing curated database of human transcripts, and GenBank accession KY503218-KY5808060 containing human breakpoint junction sequences. The unaligned sequences were then extracted by Samtools and mapped against microbial reference database using the ultrafast Karen2 algorithm (22). The microbial reference database contains 83,212 genomes, which are involved in almost all of known fungal, bacterial, archaeal, and viral genomes. Finally, the assigned taxa were aggregated into the genus level for follow-up analysis.

### Microbial Profile Analysis

Core taxa were defined as that identified at a minimum positive detection rate, present in the majority of the population. The positivity detection rate was set as 0.2%, and the prevalence was set as 20%, the same as the study dose (13). Alpha diversity (Shannon index) and beta diversity were calculated using vegan R-package (version 2.5–7). The microbiome analysis was conducted using microbiome R-package (version 2.1.28). Visualization was performed with R package VennDiagram (version 1.6.20) and ggplot2 (version 3.3.2). The pan-microbiome was calculated as described in the study (24).

### Statistical Analysis

Differential analysis was determined in diversity using the Wilcoxon signed rank test. PERMANOVAR was used to quantify multivariate community-level differences in microbial composition among groups. P-value <0.05 was considered significant at the group level.

## Results

### Dataset Characteristics

Four PDAC datasets with mRNA sequencing data were selected for this study (SRP096338, SRP197641, ERP022034, and TCGA-PAAD results from Poore’s study). The four datasets contained 214, 191, 30, and 147 samples, respectively. The meta information of each dataset was listed in **Table 1**, which contained some biological and technological factors that might have effects on the composition of microbial reads in sequencing data. It is obvious that each dataset has a special emphasis. The study SRP096338 focuses on the sampling site, which used laser capture microdissection to obtain epithelium and stroma samples from human pancreatic ductal adenocarcinoma frozen sections. And the study SRP197641 contains the RNA-Seq data from two sources, including human PDAC tissue and PDX samples. The samples of study ERP022034 were all derived from PDAC xenograft samples collected from the nude mouses. The samples of the TCGA-PAAD study are labeled with gender and age information. These data will provide us with an effective way to explore the microbial composition of PDAC microenvironments and associations with the above-mentioned factors.

### Validation of Computational Framework

Before the analysis of the above PDAC datasets, two additional datasets were selected to test our computational framework. Dataset GSE105083 was derived from a study of HPV-positive and negative head and neck cancers (25), which was used as a positive control. Dataset GSE74927 was derived from a study of PDAC cell line research with drug intervention (26). The cell lines were purchased from the commercial company ATCC, which should not have the microbial organisms, and was used as a negative control.

**Figure 1:**
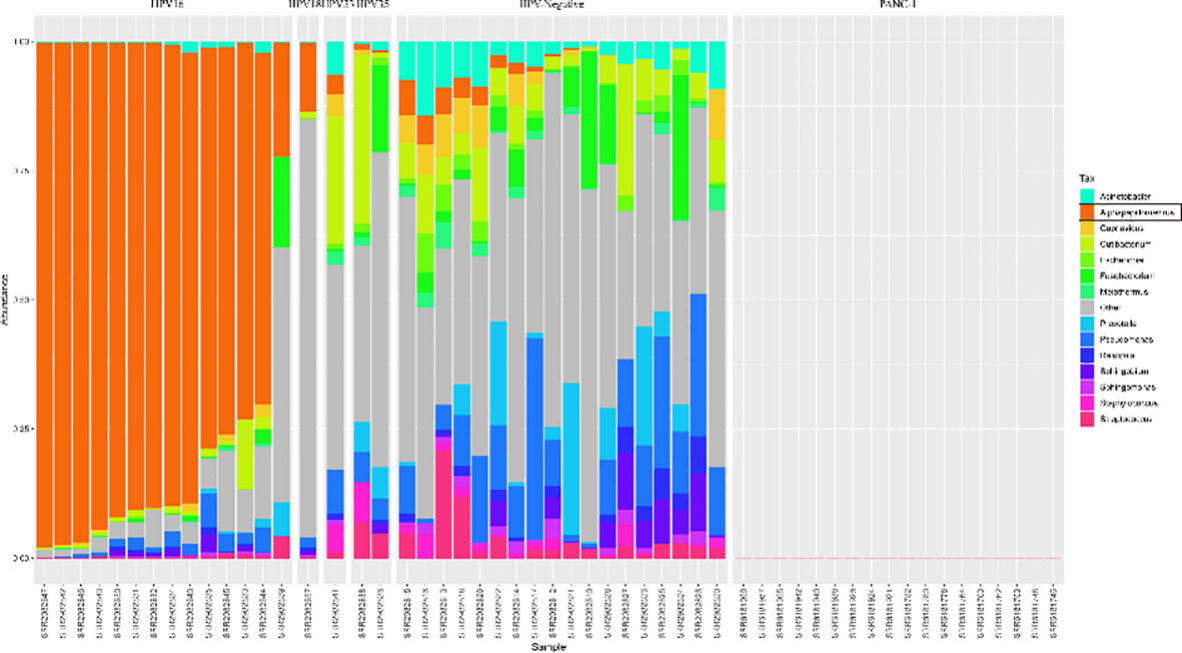
Distribution of microbial relative abundances at the genus level in the Head and Neck cancer and Panc-1 cell line datasets. The hpv (human papillomavirus) status is labeled at the top. The samples are sorted by the relative abundance of genus *Alphapapillomavirus*, which is colored by orange. The top 14 abundant genera are presented, and the rest are classified as Others.

The reanalysis results were shown in **Figure 1**. The HPV+ samples were all found to have reads in genus *Alphapapillomavirus*. Its relative abundance in HPV16+ samples is higher than that in HPV18+/HPV35+/HPV33+ samples. Interestingly, some HPV− samples were also found to have *Alphapapillomavirus* reads with a lower relative abundance. The reason for the inconsistencies could be due to the fewer detected *Alphapapillomavirus* reads, which are less than 500, the threshold set in the original study (25). In addition to viral taxa, some bacterial taxa were also identified in head and neck samples, especially higher in the HPV− samples. For cell line mRNA data, no microbial reads were identified in each sample. Therefore, quite a few microbial sequences existed in the tumor RNA-seq data, and our computational framework is reliable and stable in identifying microbial reads.

### Microbial Presence in PDAC Transcriptome Data

**Figure 2:**
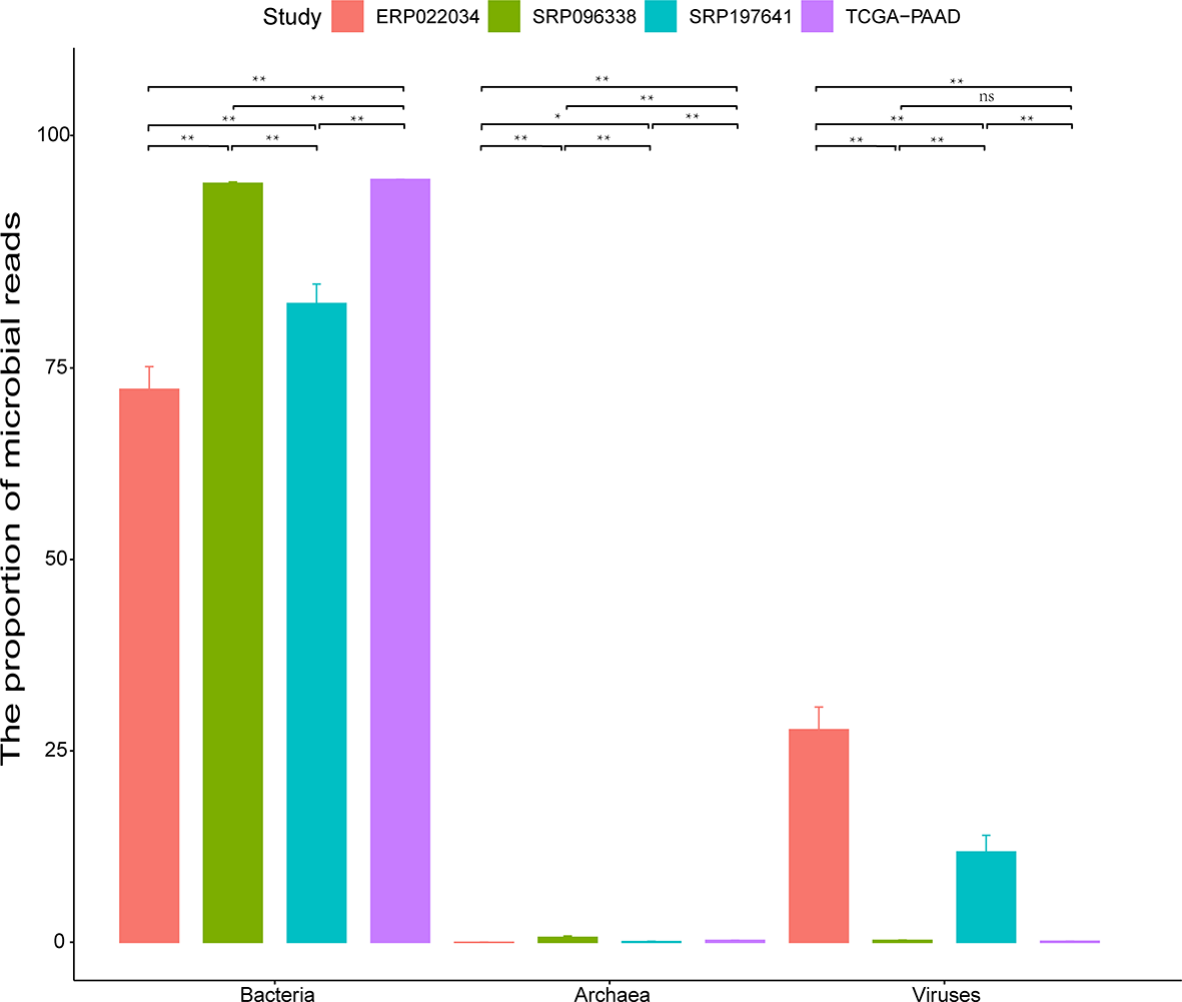
The proportion (%) of bacteria, viruses, and archaea in identified microbial reads in each dataset. The standard deviation is plotted on the graph. Statistical analysis is conducted using the Wilcoxon signed rank test. The sign ‘**’ means p-value <0.01, and ‘*’ means p-value <0.05. ‘ns’ means Not Significant.

The microbial profiles detected from the three selected PDAC datasets with tissue samples were generated using the computational workflow. Microbial reads were detected across most of the samples as shown in **Table S1**. Nine samples were found to have no microbial reads. Another 33 samples had microbial reads less than 100. Interestingly, most of these samples were all derived from biopsy, suggesting that sampling mode might have a great effect on estimating the microbial composition of tissue microenvironments. The bacterial and viral reads make up the majority of microbial reads, while the archaeal reads take a negligible proportion (**Figure 2**). It is clear that the datasets TCGA-PAAD and SRP096338 have a higher bacterial proportion than the datasets SRP197641 and ERP022034 as a whole. In contrast, an obviously higher proportion of viral reads in the microbial reads in SRP197641 and ERP022034 was observed. There might be bacterial shits in dataset SRP096338 compared to SRP197641 and ERP022234, which corresponds to fresh frozen tissue samples, biopsies, and xenograft samples, respectively. In a word, there results suggested that there indeed are microbial organisms located in the microenvironment of pancreatic tumor tissues.

### More Microbes Than Thought Found in PDAC Tissues

**Figure 3:**
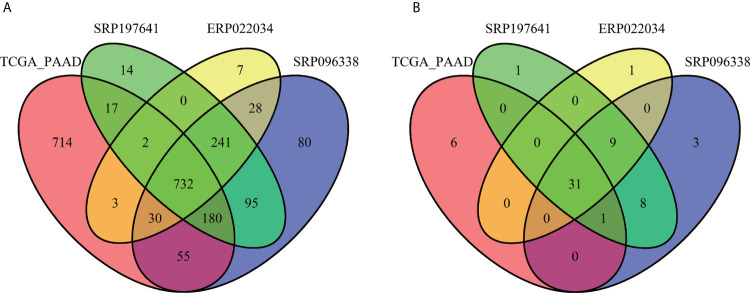
Overlap of the microbial profiles across the four datasets at the genus level **(A)** and at the phylum level **(B)**.

**Figure 4:**
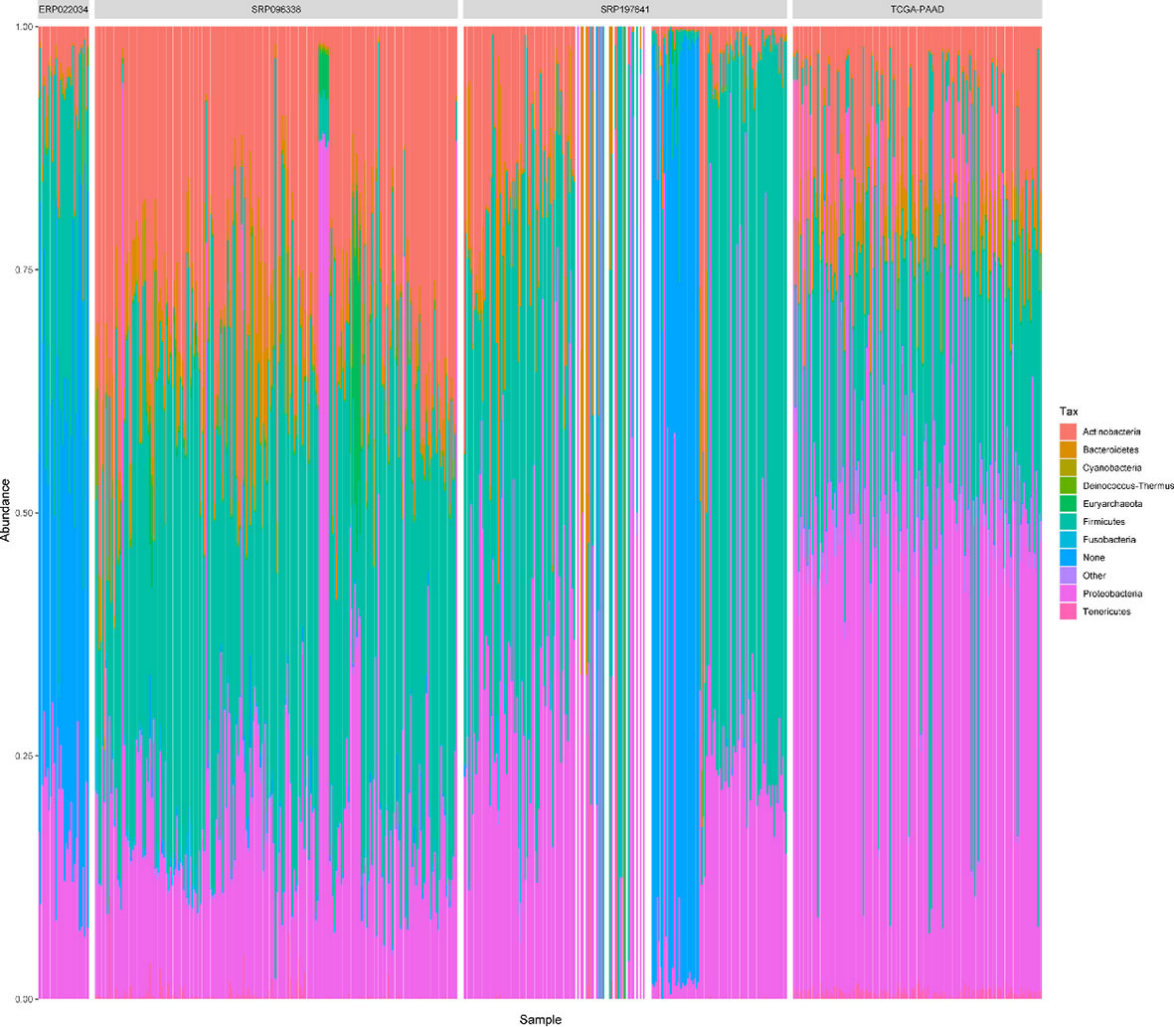
The relative abundances of top 10 phylum in all samples across the 4 datasets. “Other” represents a collection of the taxa with lower relative abundances. ‘None’ represents the taxa that has no assignment at the phylum level.

For the sake of analysis, all the taxa were assigned at the genus level. A total of 2,198 unique genus were detected across all samples, of which the four datasets contained 1,733, 1,441, 1,281, and 1,043 taxa respectively. The number of taxa in each sample across the four datasets varied greatly, ranging from dozens to hundreds as shown in **Table S2**. It is clear that there are more microbial taxa in human tissues than in PDX samples. A simple comparison was conducted among the four datasets (**Figure 3A**). From these, more than 42% (732/1,733) of the microbial composition in each dataset were shared, suggesting a relatively conservative and stable microbial composition of PDAC microenvironments. At the phylum level, the dataset SRP096338 had the most taxa, the majority of which were shared by the other datasets (**Figure 3B**). The relative abundances of the top10 phylum were shown in **Figure 4**. The total microbial composition in the four datasets was presented in **Figure S1**. Firmicute, Proteobacteria, Actinobacteria, and Bacteroidetes were the most prevalent and abundant phylum across the four datasets (**Figure S2**). Moreover, major variation in the microbial profile was driven, as expected, by the study (**Figure 4**). Inter-individual differences in taxonomic profiles did not exceed those induced by different data sources.

To identify differences and commonalities of genus shared between and cross each dataset, core microbiota was characterized. 56, 54, 20, and 22 genera were respectively identified as core microbiota in each dataset. 54 core microbiotas were identified across the four datasets. Among them, 8 genera: *Kocuria*, *Streptococcus*, *Bacillus*, *Lactobacillus*, *Ralstonia*, *Staphylococcus*, *Acinetobacter*, *Pseudomonas* were shared. However, the relative abundances of the genera varied greatly across the 4 datasets (**Figure S3**).

**Figure 5:**
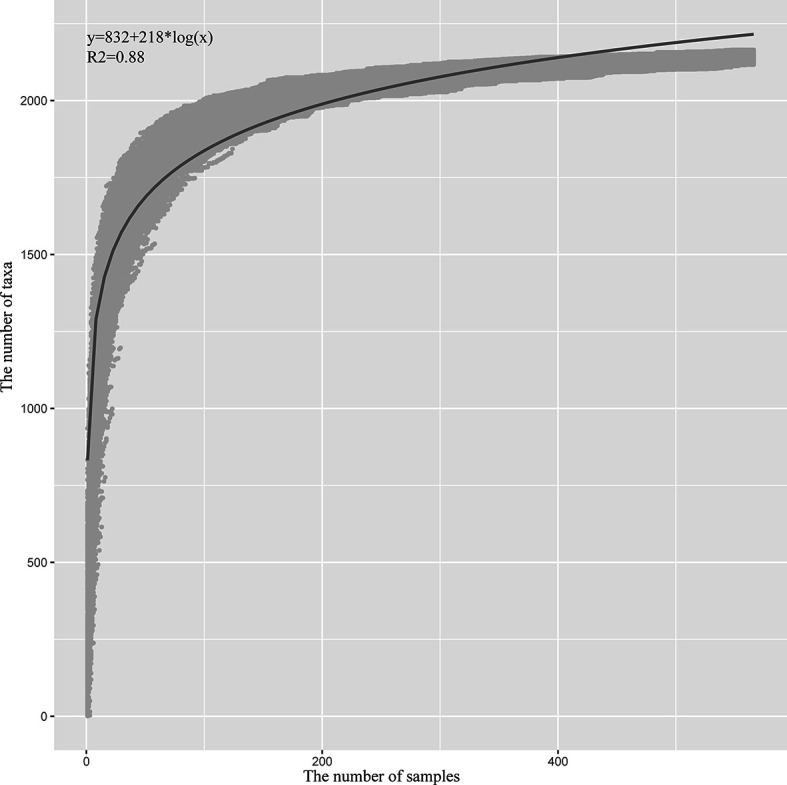
Statistic estimation of the size of pan-microbiome in PDAC tissue. The x-axis is the number of samples, and the y-axis is the cumulative number of taxa. The process is briefly described as follows: One sample is randomly selected at a time, and the cumulative number of taxa is counted until 582 samples are obtained; then the process is repeated 1,000 times. All the data points are indicated in gray, and the blue line is the fitted curve. The fitting formula and R2 value are labeled at the top-left corner.

To estimate the number of microbial taxa at the genus level in PDAC tissue, the concept of “Pan-genome” was referred (27). Even with the inclusion of 582 samples, the pan microbial profile in PDAC tissue appears not to have been reached, as depicted in the accumulation curve (**Figure 5**). We estimated the pan microbiome of PDAC probably surpasses 2,500 taxa. Therefore, there are more microbes than thought in PDAC tissues, which should get more attention in future research studies.

### Factors Affecting the Microbial Diversity and Composition in PDAC Tissues

Alpha diversity (Shannon index) and beta diversity were calculated to estimate the microbial diversity of tumor microenvironments. Among the four datasets, both the Shannon diversity and beta diversity showed significant differences between any two groups. Moreover, diversity varied by sex, age, sampling site, sample source at varying degrees in different datasets. We compared the microbial relative abundances and diversities in each dataset with the emphasized factors as described in *Methods*.

#### Gender

The datasets ERP022034 and TCGA-PAAD have the gender phenotype for each sample. The gender group (female *vs*. male) had no significant effect on the overall microbiota composition in either of the datasets (PERMANOVA test, p-value = 0.332 for ERP022034 and 0.263 for TCGA-PAAD). The same results were observed in Shannon diversity between the female and male samples. However, the divergences within the female and male samples with respect to the median profile with each group were calculated, and the male group was found to have significant higher values than the female group in TCGA-PAAD (**Figure S4A**). The results suggested that although there were no differences in microbial composition between female and male samples as a whole, the male group has a more heterogeneous community composition.

#### Age

The dataset TCGA-PAAD has the phenotype of age at diagnosis for each sample. In order to explore the effect of age on microbial composition, we divided the samples into two groups: Old (>65 year old), and Young (<=65 year old). PERMANOVA analysis showed that the age group (Old *vs*. Young) also had no significant effect on the microbiota composition (p-value = 0.727). However, different thresholds ranging from 45 to 70 were set to get the age groups, and there were still no significant differences between the Old and Young group. These results suggested that the microbial composition might be in a stable state after tumor initiation and progression. As for diversity, there were no differences in Shannon index between the Old and Young groups (Wilcox test, p-value = 0.069), but there were significant differences in divergences within the two groups (**Figure S4B**). The microbial composition within the samples of the old group varied more greatly than that within the samples of the young group. In this way, the patients with older age would have a more heterogeneous tumor microbiome in the PDAC tissue.

#### Sampling Site

As we know, the tumor microenvironment is complex, which contains tumor cells, normal cells, immune cells, neutrophils, and so on. The tumor microenvironment is also not confined to a particular region, including stroma, epithelium. It is necessary to explore whether the different sites of tumor tissue have an effect on microbial composition.

**Figure 6:**
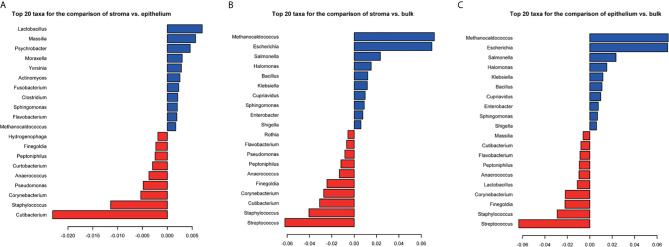
The coefficients of top 20 taxa separating the stroma and epithelium samples **(A)**, the stroma and bulk samples **(B)**, and the epithelium and bulk samples **(C)**. PERMANOVA significance test is used for group-level differences. Red represents the negative coefficients and blue represents the positive coefficients.

The dataset SRP096338 contained samples derived from the stroma, the epithelium, and bulk. PERMANOVA analysis showed that there were significant differences among the microbial composition of stroma, epithelium, and bulk samples (p-value = 0.001 for any two groups). The top 20 taxa that separate the groups were listed in **Figure 6**. Nine genera: *Methanocaldococcus*, *Staphylococcus*, *Finegoldia*, *Corynebacterium*, *Anaerococcus*, *Peptoniphilus*, *Flavobacterium*, *Cutibacterium*, and *Sphingomonas* were shared among the three sets of significant different genera. As for diversity, there were significant differences in Shannon index and divergence between the stroma and epithelium groups (**Figure S5**). The Shannon index and Inter-individual divergence in the epithelium group were both higher than that in the stroma group. In this way, the result suggested that the different sites of tumor microenvironment indeed have an effect on microbial composition.

#### Tissue Source

The dataset SRP197641 contained samples from human tumor tissues and PDX samples, which could be used to detect whether there is a difference in microbial composition of the tumor tissue and PDX samples. In order to reduce the effects of technical factors (SequencingPlatform, ExperimentStrategy, and LibraryLayOut) on microbial composition, 70 samples with the same platform, ExperimentStrategy and LibraryLayOut were selected. There were significant differences in microbial composition between the human tumor tissue and PDX samples (PERMANOVA, p-value = 0.001). The top 20 taxa separating the group were listed (**Figure S6A**), of which *Gammaretrovirus* and *Ralstonia* were the most coefficient taxa with PDX samples and human tissue samples, respectively. Moreover, 17 of the 20 taxa were found to show significant differences in relative abundance between the datasets SRP096338 and ERP022034, which correspond to a set of human tissue samples and PDX samples, respectively. The relative abundance of *Gammaretrovirus* in ERP022034 was 376 times higher in average compared to that in SRP096338. Other taxa *Staphylococcus*, *Ralstonia*, *Moraxella*, and *Cloacibacterium* also showed higher abundances in the PDX samples. The left genus: *Corynebacterium, Streptococcus, Bacillus, Pseudomonas, Acinetobacter, Salmonella, Massilia, Lactobacillus, Halomonas, Pantoea*, and *Clostridium* were highly abundant in the human tumor tissue samples. As for diversity, the human tissue samples had significantly higher values of microbial richness and divergence than PDX samples did (**Figure S6**).

## Discussion

A number of studies have provided evidence supporting a microbiome–pancreas axis for diseases originating in the pancreas with a focus on PDAC. However, a number of questions remain unanswered, such as the exact composition of tumor microbiome in PDAC. Although the Human Microbiome project publishes the microbial landscape of some tissues or organs (28), like gut, oral, vagina, and skin, there is little data on vital organs and tissues in the body, such as the pancreas. In this study, we integrated four datasets to explore the composition of PDAC microbiome and further analyze whether some biological factors have effects on the composition. The results would be helpful for the microbiome–pancreas axis related research studies.

The kinds of microbes in PDAC tissues are more than what they are thought to be. A total of 2,198 genera were found across the four datasets, and 313 genera in average were identified in each sample. The pan-microbiome was estimated to be open, the size of which might surpass 2,500. However, the core microbiota of PDAC tissues takes a small proportion, which covers a few dozen genera. Only eight core genera were shared across the four datasets, which indicates that there are large differences among PDAC tissues. Among them, *Streptococcus, Bacillus, Staphylococcus*, and *Pseudomonas* were detected in Erick’s study, which characterizes the microbial profile of PDAC tissue samples *via* the 16S rRNA gene sequencing (10). *Acinetobacter* was also reported in PDAC in another study (3). Interestingly, *Acinetobacter, Staphylococcus*, and *Pseudomonas* are the three most common concurrent infecting organisms (29). *Kocuria* spp. are non-pathogenic commensals of the skin, mucosa, and oropharynx and are also found to cause both superficial and deep-seated/invasive infections involving both immunocompromised as well as immunocompetent individuals (30). The coexistence of these pathogens or opportunistic pathogen suggests an infected state of PDAC tissue and a potential anti-infection treatment for PDAC. *Lactobacillus* was reported to attenuate the progression of pancreatic cancer promoted by porphyromonas gingivalis in Kras G12D transgenic mice (31). Ralstonia is one of the emerging pathogens prevalent in the airways of individuals and keeps an increasing trend (32). It is also prevalent in oral cancer (33). Its association with PDAC has not been reported and needs to be further studied. From this perspective, the core or prevalent taxa distributed among PDAC tissues should be paid more attention in future research studies, which might play important roles in the tumorigenesis of PDAC.

Unexpectedly, the biological factors (gender and sex) have no significant effects on the composition of tumor microbiome in PDAC. The only remarkable result is that the divergence of microbial composition among the samples in the male group is higher than that in the female group. Similar results are also observed in the comparison of Age and microbial composition in PDAC. Older PDAC patients have more divergent microbial profiles. These results suggest that the tumor microbiome might be in a stable state when the tumor occurs. However, in most studies, gender and age are reported to be risk factors of pancreatic diseases (34). Therefore, it is necessary to explore the microbiome of pancreatic tissue in healthy individuals and the changes between healthy and ill individuals.

As we know, the tumor microenvironment is a complex integrated system, which mainly composed of multiple regions, like stroma, epithelium, micro vessel, and so on. Some important processes happen in these local regions. For example, stroma is a potential place for targeted drugs to act on tumor cells, which could improve anti-cancer drug efficacy (35, 36). The microbiome of the tissue microenvironment has a great effect on drug therapy. Therefore, it is necessary to further focus on the microbiome of local regions of PDAC tissue. Our results found that there are significant differences in tumor microbiome of PDAC between stroma and epithelium sites. The genus *Staphylococcus, Finegoldia, Corynebacterium, Anaerococcus, Peptoniphilus*, and *Cutibacterium* were found to have significantly higher relative abundances in stroma compared to epithelium, and *Sphingomonas* and *Flavobacterium* were found to have an opposite trend. This phenomenon should get more attention in tumor microenvironment related studies. It also suggests that the microbial composition in these more focused tissue regions and their impacts on the tumorigenesis should be explored separately in subsequent research studies.

Similar results were also observed in microbial composition between tissue sample in human and PDX samples in mice. It is the first time to explore the presence of microbes in PDX samples. The PDX samples have a lower microbial richness and inter-individual divergences compared to human tissue samples, suggesting that tumor xenotransplantation has an effect on the tumor microbiome. Several genera were filtered out with remarkable changes in relative abundances. Among them, the genus *Gammaretrovirus* with the most dramatic change was reported to be mouse-xenotropic (37). *Ralstonia pickettii* was identified as the causative agent of the ataxia syndrome in immunodeficient mice (38), suggesting a possible enrichment of *Ralstonia* spp. in PDX mouse. Whether these microbial changes have any effects on the biological characteristics of PDX samples compared to primary tumor samples needs further research. For example, the common application of PDX models in drug screening should pay more attention on the microbial changes.

However, this study has serval flaws. First, the microbial composition was not detected based on traditional methods, like 16S rRNA sequencing or qPCR. The microbial profile identified for each sample could have a bias, since the RNA-Seq data has library artifacts (38). Second, the significantly different genera were not validated in real samples using the traditional methods. Finally, microbial contaminants were not analyzed and removed in the above analysis. The significantly different genera might contain exogenous contaminants.

Despite the above flaws, the method of microbial profiling based on RNA-Seq data was validated in other studies (13, 15, 39). We also validate our computational framework using two public datasets. Therefore, the above results are reliable to some extent. In addition, the purpose of this study is not to determine the detailed microbial composition of PDAC tissue samples but to provide an overall landscape of tumor microbiome of PDAC and its association with some important biological factors. In this perspective, our study achieved its purpose.

## Conclusion

As far as we know, no studies were conducted to systematically explore the microbial composition of tumor microenvironment in PDAC tissue samples and its association with other biological factors. In this study, the landscape of microbial composition of PDAC tissue through integrating four public datasets is presented for the first time. The number of microbes located in each PDAC tissue sample and the number of microbes in PDAC microbiome were both estimated, which provides a quantitative understanding of tumor microbiome in PDAC. 8 core microbiotas (*Kocuria, Streptococcus, Bacillus, Lactobacillus, Ralstonia, Staphylococcus, Acinetobacter*, and *Pseudomonas*) were identified to be prevalent and abundant in and across the 4 datasets, which should be paid more attention. Moreover, gender and age are found to have no significant effect on tumor microbiome, but sampling site and tissue source do. These results play important guiding roles in the follow-up studies of tumor microbiome, such as the role of intra-tumor bacteria on anti-tumor immunity. Certainly, the above results need to be validated in real samples, and more work needs to be continued.

## Data Availability Statement

The original contributions presented in the study are included in the article/**Supplementary Material**. Further inquiries can be directed to the corresponding authors.

## Author Contributions

DY conceived and designed the study. DY, TW, and DL performed all the data analyses. YM and TW helped the preparation of figures and tables. DY, WZ, and SG contributed to the writing of the manuscript. All authors contributed to the article and approved the submitted version.

## Funding

1. The Shanghai Science and Technology Commission Sailing Program (NO.: 20YF1458100). 2. The School-level Project (No.: 2018QN02) in the Second Military Medical University. 3. Shanghai Key Laboratory of Cell Engineering Program (NO.: 14DZ2272300).

## Conflict of Interest

The authors declare that the research was conducted in the absence of any commercial or financial relationships that could be construed as a potential conflict of interest.

## Supplementary Material

Supplementary Table 1 The number of identified microbial reads in each sample across the four PDAC datasets.

Supplementary Table 2 The number of identified genus in each sample across the four PDAC datasets.

Supplementary Table 3 The relative abundances of 732 genera shared by the four PDAC datasets.

Supplementary Table 4 The list of core microbiotas characterized in and across the four PDAC datasets. The bold words correspond to the genus shared by the five sets of core microbiotas.

Supplementary Figure 1 Microbial composition of PDAC tissue samples across four datasets at the phylum level.

Supplementary Figure 2 The relative abundances of the four phyla across four datasets. Each phylum is presented in a separate frame.

Supplementary Figure 3 The relative abundances of eight core genera in the four datasets. Each genus is presented in a separate frame.

Supplementary Figure 4 The divergence distribution within the gender groups **(A)** and the age group **(B)**. The Wilcoxon signed rank test was used for the statistical differences. P-values are labeled.

Supplementary Figure 5 The distribution of Shannon index and divergences of each sample within the groups (stroma, epithelium, and bulk). The Wilcoxon signed rank test is used for the statistical differences. P-values are labeled.

Supplementary Figure 6 The top 20 taxa with significantly different relative abundances **(A)**, Shannon index **(B)** and divergences **(C)** between the groups (human tissue *vs.* PDX), The Wilcoxon signed rank test is used for the statistical differences of divergences. P-values are also labeled.
