# Supplementary figures and images for: The Landscape of Microbial Composition and Associated Factors in Pancreatic Ductal Adenocarcinoma Using RNA-Seq Data
Source: Front Oncol. 2021 May 31;11:651350. doi: 10.3389/fonc.2021.651350 (PMC8202409; doi:10.3389/fonc.2021.651350)

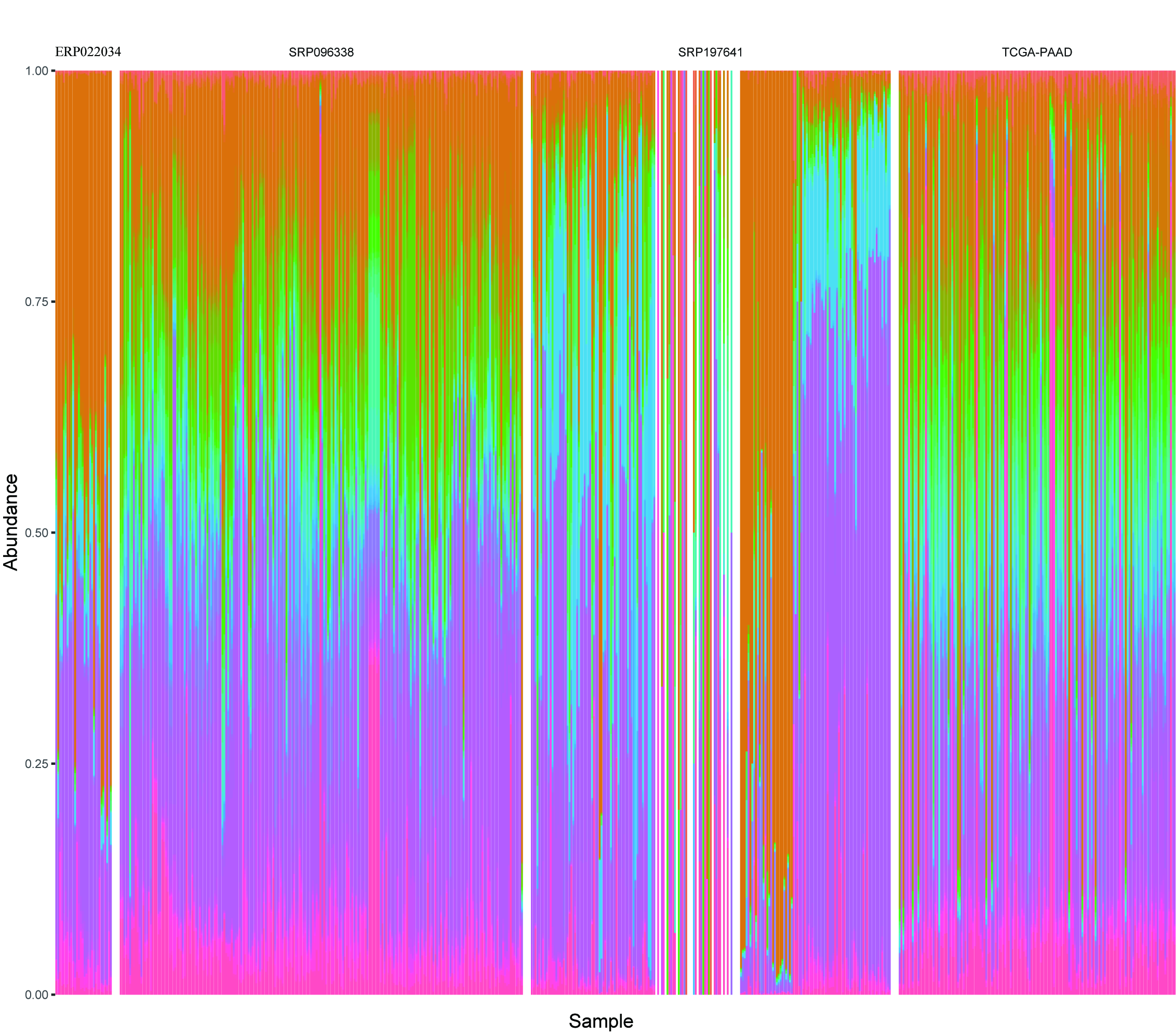

Supplement: Supplementary Figure 1 — Microbial composition of PDAC tissue samples across four datasets at the phylum level. [file Image_1.tif]

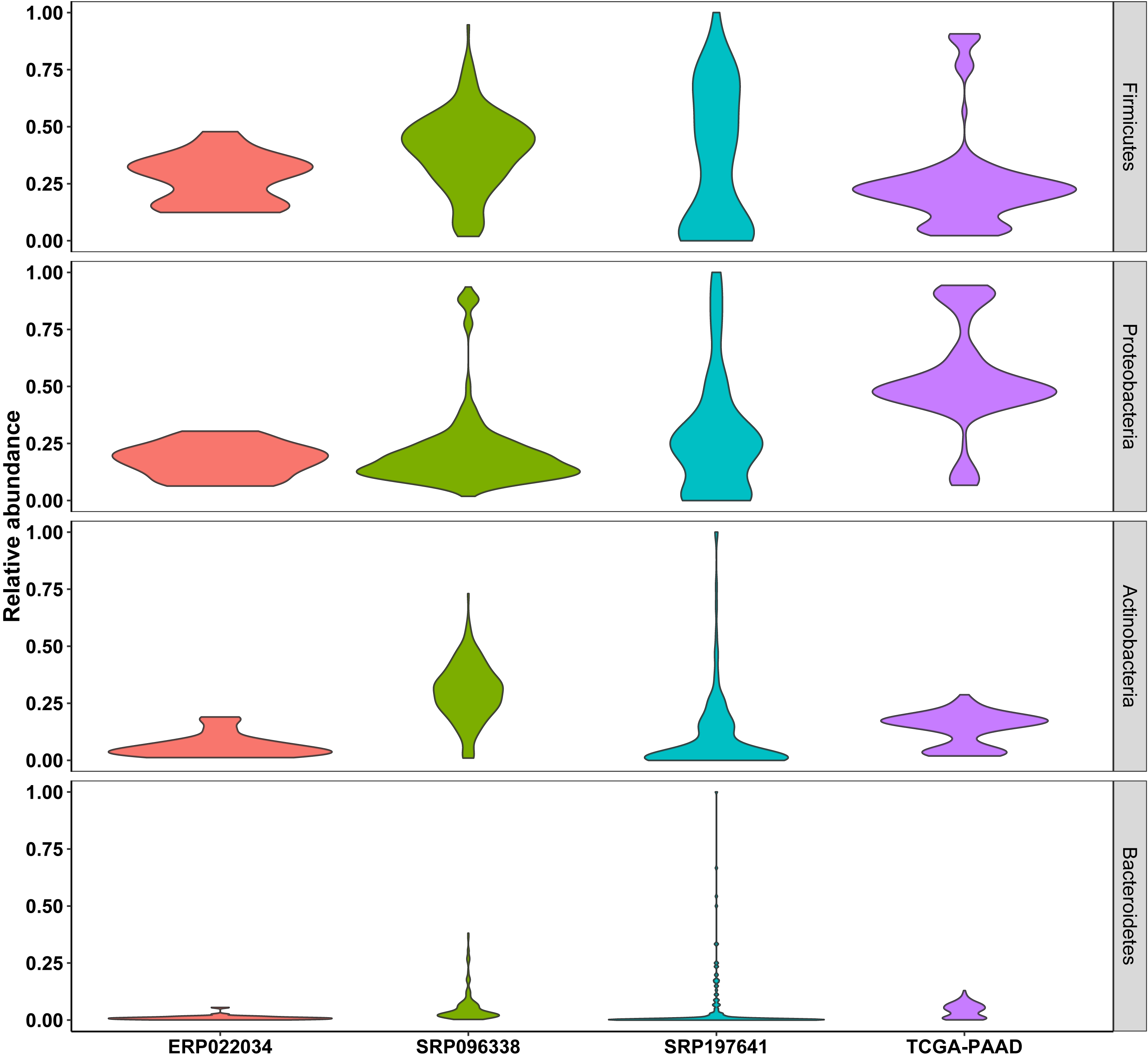

Supplement: Supplementary Figure 2 — The relative abundances of the four phyla across four datasets. Each phylum is presented in a separate frame. [file Image_2.tif]

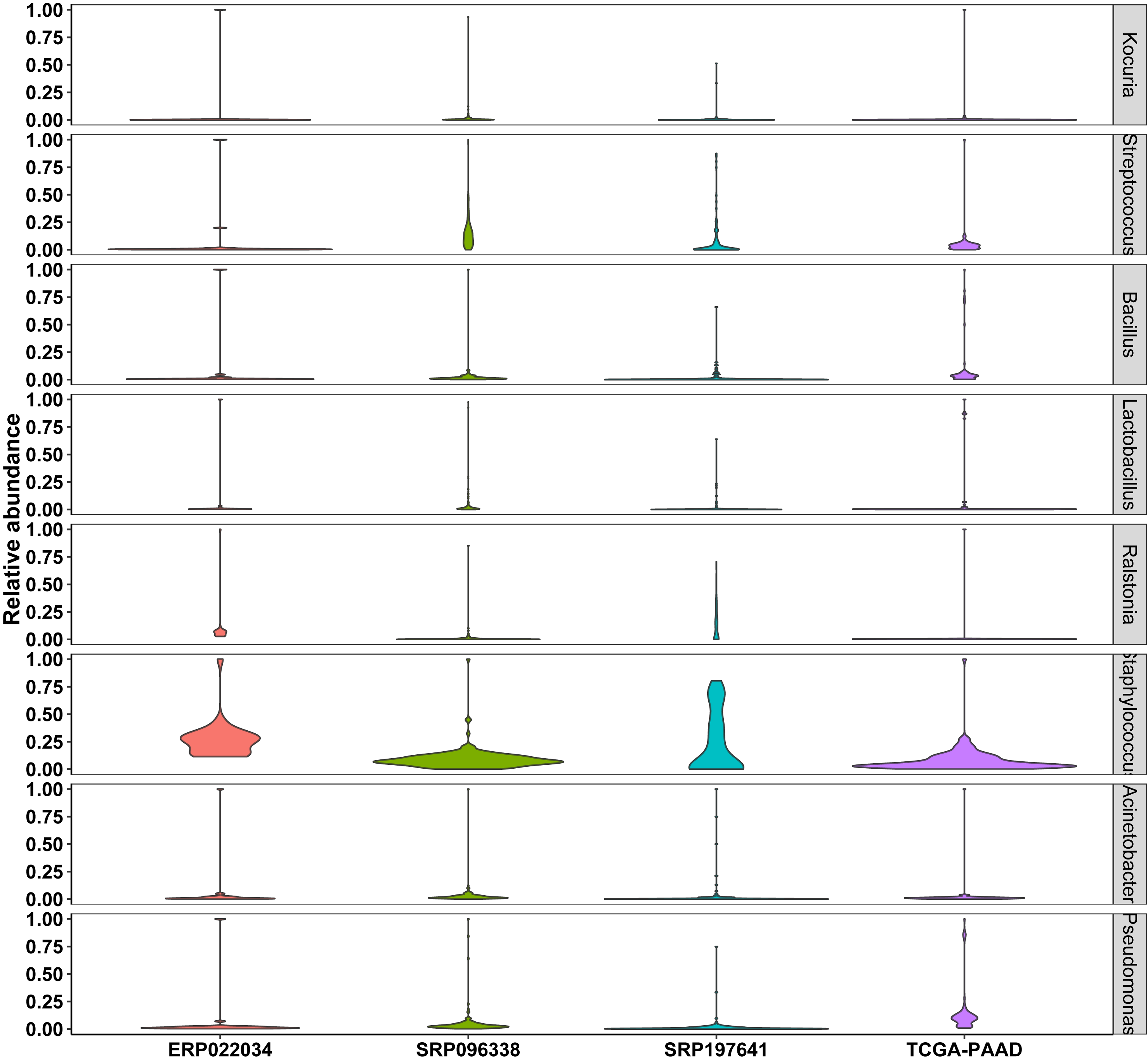

Supplement: Supplementary Figure 3 — The relative abundances of eight core genera in the four datasets. Each genus is presented in a separate frame. [file Image_3.tif]

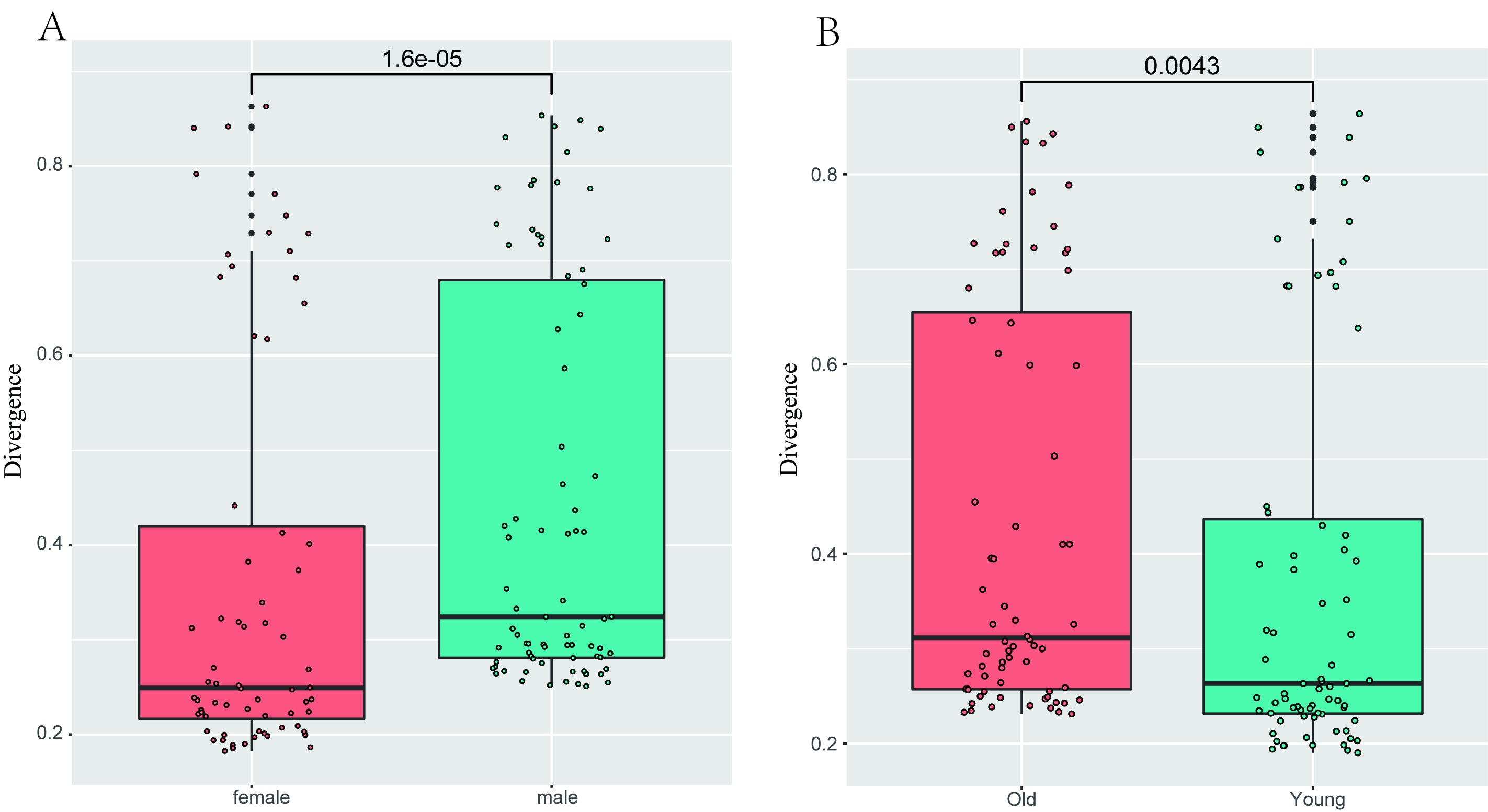

Supplement: Supplementary Figure 4 — The divergence distribution within the gender groups (A) and the age group (B). The Wilcoxon signed rank test was used for the statistical differences. P-values are labeled. [file Image_4.tif]

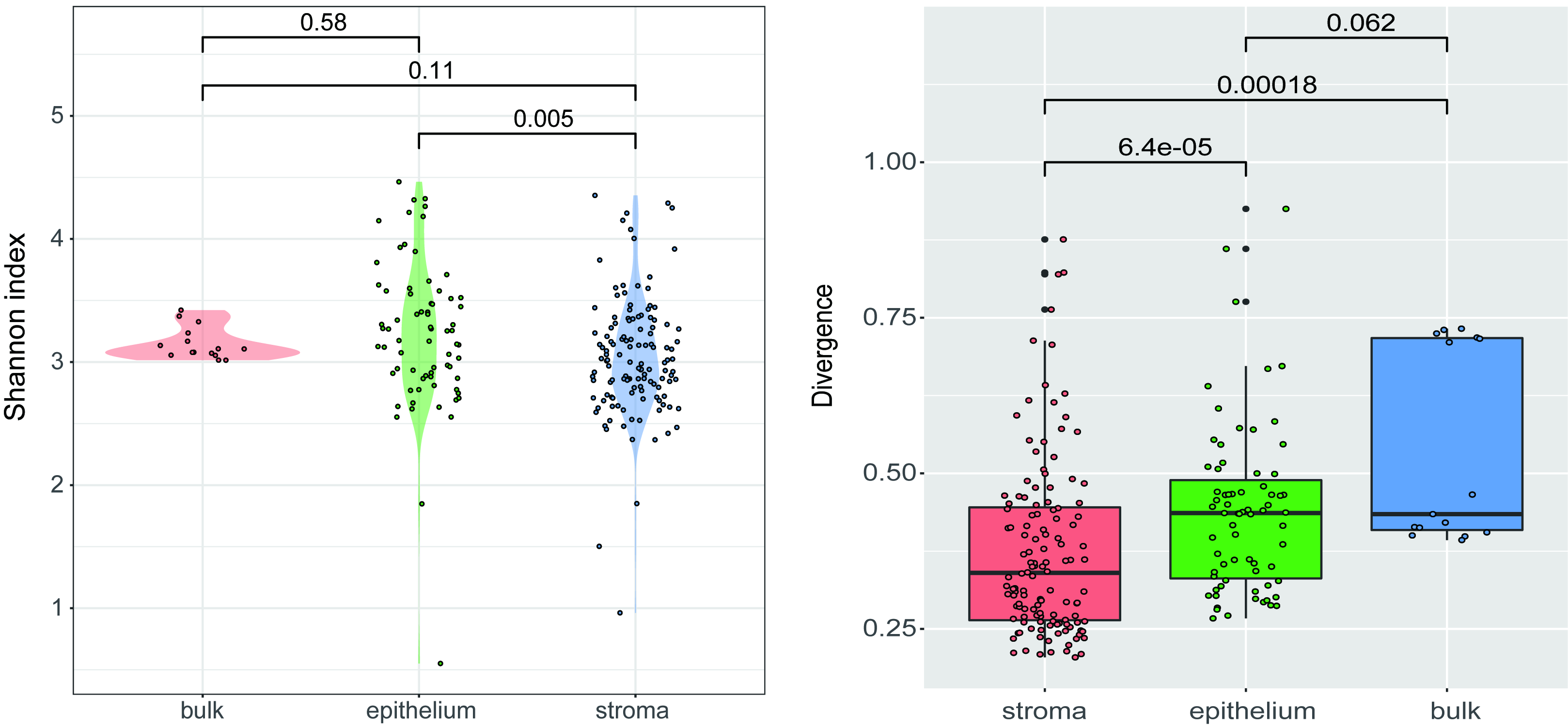

Supplement: Supplementary Figure 5 — The distribution of Shannon index and divergences of each sample within the groups (stroma, epithelium, and bulk). The Wilcoxon signed rank test is used for the statistical differences. P-values are labeled. [file Image_5.tif]

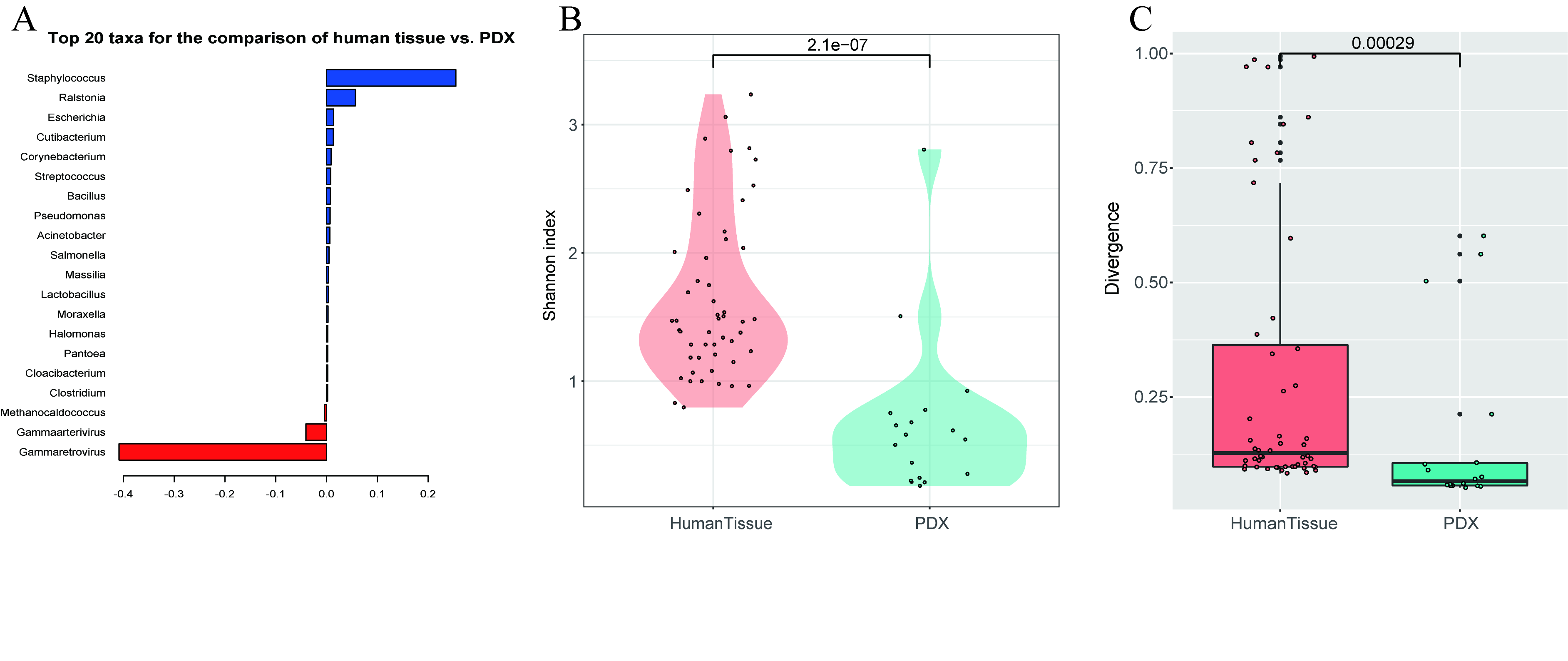

Supplement: Supplementary Figure 6 — The top 20 taxa with significantly different relative abundances (A), Shannon index (B) and divergences (C) between the groups (human tissue vs. PDX), The Wilcoxon signed rank test is used for the statistical differences of divergences. P-values are also labeled. [file Image_6.tif]
